# What makes it likeable? A study on the reactions to messages in a digital social network: the case of Facebook in Farsi

**DOI:** 10.1186/s40064-016-3771-3

**Published:** 2016-12-20

**Authors:** Shaho Sabbar, Daiwon Hyun

**Affiliations:** 1 University of Tehran, Tehran, Iran; 2 Sogang University, Seoul, South Korea; 3 Faculty of World Studies, Northern Amir-Abad, 15th & 16th streets, Tehran, Iran

**Keywords:** Flow of information, Network behaviors, Social network, Message, Reactions, Facebook

## Abstract

**Background:**

After a piece of information is put into a network, its fate depends on the behaviors of the nodes of the network; nodes that are equipped with the hardware and software of the age of information and are more powerful than any time in the past. This study suggests that a useful research for communication, marketing and advertising would be one that looks for patterns in the reactions of the nodes toward different pieces of information.

**Results:**

This study has used Facebook to see how people have reacted to different types of messages in terms of liking, sharing and commenting. Rather than looking for universal, generalizable patterns we have tried to examine the practicality of the proposed method. The practical aspect of the study comes after a short theoretical discussion on the issue of flow of information in a digital world. The results revealed dozens of significant relations between the examined variables.

**Conclusions:**

This study, its theoretical discussion and results suggest that it would be practical to study the relations between the characteristics of Facebook messages and the type of reactions (liking, sharing and commenting) that they attract.

## Background

### The age of powerful nodes

Today social media are ubiquitously used by individuals (Peters et al. 2013) and the internet is considered as an important source of growth for many companies (Hoffman and Novak 2009). More than 70% of organizations around the world used social media in 2011 (KPMG 2011) and now nearly 90% of U.S. companies use them (eMarketer 2014). Internet as a cheap and accessible tool is increasingly becoming a source of information (Ratchford 2015).

Moreover, marketing strategy has experienced significant changes by social media (Varadarajan and Yadav 2009). Websites such as Facebook, Linkedin, and MySpace have attracted the attention of marketers and more attention is dedicated to computer-mediated social environments (aka CMSEs) (Yadav et al. 2013). However companies might be still experiencing how to effectively use social media for their benefit (Shankar and Batra 2009). In fact, we haven’t been able to fully utilize the potentials of the new environment (Rydén et al. 2015) and regarding flow of information on Social Media, we have only started to understand the existing patterns and processes (Hennig-Thurau et al. 2010).

What is important for marketers here is people engagement (Pan and Chiou 2011; Schamari and Schaefers 2015; Deightona and Kornfeld 2009). After a message is released in a network by an original distributor (e.g. a mass medium) it’s up to the nodes of the network how to react to it. The collective network behavior of the nodes determines where the message goes, how far it goes, and how long it stays. The spread of information by online word-of-mouth is an example of how information flows as a result of the actions of the nodes of a network (Berger and Iyengar 2013). In online and offline environments, many scholars who study word-of-mouth, fundamentally, work on how the actions of the nodes define the fate of pieces of information (Saenger et al. 2013; Heung 2008; Eisingerich et al. 2014).

The situation in which the nodes have the main role in the network flow of information is not new. It can be argued that when it comes to *network* flow of information, even in the offline world, nodes have always been the main actors. However, in a digital social network and an internet-enabled society the nodes have more power and a crucially more important role. Compared to the pre-internet times, the average node has links to a significantly higher number of nodes and its capacity of delivering messages in terms of number and volume has multiplied. Although some nodes may be more active and some may act as passive observers (Chandrashekaran et al. 2010).

Study has addressed the changed level of power of the individual actors in the network of audiences and consumers (Van Laer et al. 2013; Singh and Sonnenburg 2012; Gensler et al. 2013). While some have seen the emerging consumer power as a threat to companies and organizations (Hautz et al. 2014; Van Noort and Willemsen 2011) it is usually considered as an opportunity for businesses that can benefit from consumer engagement.

### The age of re-distribution

Study has vastly addressed the abovementioned change (from the internet-free to the internet-enabled society), but another crucial change has happened in the dynamics of flow of information that has received less scholarly attention. In the past, after the initial transmission of a message, what would flow in the society would have been the *perceptions* of the nodes. One can imagine that in rare cases a person would read a story in a newspaper and make several photocopies of it and start giving the copies to others. However, the common scenario would be different: a person would read a story in a newspaper and start *telling* others about it. This is what long ago Lazarsfeld et al. (1944) explained as the two-step flow of information.

In fact, in the pre-internet era, if the dissemination of information had two stages, the first being media-to-people and the second being people-to-people or word-of-mouth (Batinic and Appel 2013), in the second stage, original messages were not the main things to be handed among the nodes. In the second stage, several different *perceptions* of the messages were shared and re-shared. In the time of the internet-based social networks this doesn’t seem to be the case. The technologies of the age of digital social networks and especially the explosively fast spreading mobile devices (Shankar et al. 2010) have enabled the *re*-*distribution of the original messages* in a massive scale.

If you are a member of a cellphone messaging system such as Viber, WhatsApp, Telegram, Kakao or the like, you may receive, on a daily basis, several pieces of audio, video, picture and text that were cut from a bigger content, previously published. These pieces of information may include a picture of the cover of a magazine, a piece of video cut from a TV program or a movie, an audio someone recorded at a debate at college, etc.

This adds to the importance of the role of the average members of the network as the contributors to creation and distribution of content. The number of people only in the U.S. who created content in 2013 estimated to be more than 110 million (Ertimur and Gilly 2012). Based on some estimates one out of four internet users are serious creators of content. Since in 2015 about 3.2 billion people used the internet (ITU 2015) there must have been some 800 million people who created digital content in that year. Users of Facebook alone share nearly 2.5 million pieces of content and email users send over 200 million messages per minute (Gunelius 2014).

The abovementioned changes have turned the average citizens to serious content creators and distributors (Nagao 2003). The possibility to take a message and re-distribute it in its original form, in a massive scale, is now there and it is being widely used by the normal nodes of our digital society. In this environment, the distribution of the materials produced by the media organizations and other major content creators depend on the decisions of the normal nodes of the network. We have tried in this paper to test a way of studying node decisions as a powerful force in the distribution of content in our digital network.

### Network behaviors

Social networking sites have become a new social phenomenon that “is changing the way millions of people communicate and share information with each other” (VanMeter et al. 2015: 70). As argued above, an important stage of flow of information just begins after the initial distribution by the mass media and other mass content distributors. People take pieces of information from the original sources and redistribute them through social networks. These pieces of information are detached from the whole package of information originally distributed and in some cases the original source might even be unknown to most audiences.

In this process, people get to make very different decisions and show different *network behaviors* for every single piece of information. Cut two 1-min videos from a TV program that were aired one right after another. Then share them on a digital network. It would not be too surprising if one piece attracts no attention while the other is redistributed by thousands of the network members. This study is an effort to look into *network behaviors of the nodes* as factors that define the fate of messages in a network.

The concept of network behavior is similar to that of sequencing by Ying Mo and Wellman (2012) by which they mean the process of decision-making by the nodes during the flow of information. Scholars of marketing have also addressed the concept under the term of herding (Langley et al. 2014).

In marketing, which is about exposing the right person with the right message at the right time (Vilnai-Yavetz and Tifferet 2015) the behaviors of the nodes are studied, mostly within the study of viral marketing and word-of-mouth. In this field whatever affects the flow of information is critically important. If fact, “viral marketing has become a pivotal component of marketing strategy” (Koch and Benlian 2015:37) and computer-mediated word-of-mouth is sometimes considered as the most important marketing tool (Jiménez and Mendoza 2013; Goyette et al. 2010) especially with the development of Web2 (Hausmann 2012) and the expansion of social media that have provided word-of-mouth with an unparalleled platform to function (Chen et al. 2011).

Marketers are aware of the comparative importance of volunteer act of the nodes of a network in passing pieces of information, compared to diffusion of the messages using mass media (see Kaplan and Haenlein 2011; Pescher et al. 2014; Camarero and San José 2011). They are interested in putting out a message on the network and not worrying about the costs of distributing it, hoping that people would take care of the job and make the process of marketing affordable (Aksoy et al. 2013).

It would be an ideal world for a marketer in which he or she could look at a message and know what kinds of reactions it would receive from different nodes of the network. This study is a preliminary work in this area. We have proposed an approach based on studying the relations between the characteristics of the messages and the kinds of reactions they are more likely to receive.

### How to study reactions to materials

Above, we discussed some changes in the role of the nodes of the network. We also argued that we should focus more attention on the network behavior of the nodes after they receive a piece of information. This general goal can be approached from many ways devised by the researchers. In fact the literature reviewed so far, is not specifically about the questions we are going to present in this study. In this section, we will review some studies that have asked questions related or similar to our research questions.

If the most important factor that defines the outcomes of a network is the collective network behaviors of the nodes, better knowledge and understanding about the behaviors of the nodes may enable us predict the fate of different pieces of information on the network. There is limited literature regarding the way one should systematically study the network behaviors of the nodes.

Choi and Toma (2014) in a study on Twitter posts found relations between the positivity of the posts and their likelihood to be shared. They say “highly intense positive events” had a better chance to be shared on Twitter than “low-intensity positive events”. Interestingly they were able to study face-to-face relations too and concluded that “highly intense negative events” had a higher chance to be shared face-to-face than “low-intensity negative events”.

Among different possible characteristics of online materials, Choi and Toma considered positivity and negativity and their relation to the network behaviors of the nodes (here sharing). However, there are many other characteristics that can be considered in a more detailed and comprehensive work. For instance, instead of the more general concept of positivity one could look into the materials being disappointing, nostalgic, emotional, comical, satirical, etc.

The abovementioned characteristics are mainly about the *form of expression* of the materials. However, the *subject* of materials can be also taken into consideration. In a research on Facebook Yu (2016) studied materials on Facebook and Twitter to see how political and non-political material were in relation to people’s expressions. Subject of online material can include politics, economy, psychology, medicine, science, etc.

Fleuriet (2014) and her colleagues saw the characteristics of a Facebook post as “containing text only”, “text plus either an attractive or unattractive photo of the sender”, “a winking face emoticon”, “words in all capitals”, or “triple exclamation points”. Then they studied how these different posts could affect the viewers emotionally.

In a study with a relatively similar approach to ours, Yu et al. (2011) used the same two sides of relation: the characteristics of Facebook posts—that they saw as *content* and *media type*—and the popularity of the messages—that they judged by the number of likes they received.

These studies linked the characteristics of online materials (political, positive, etc.) to how they were treated by people (shared, liked, and commented on). The two sides of relations are what the current study aims to investigate but in much more details.

Other studies have considered the characteristics of the *node* of the network. For instance Lee et al. (2014) looked into relations between personality traits such as being extravert, narcissist etc. with a series of actions on Facebook such as liking, sharing, frequency of updating their status, etc. The current study excludes the characteristics of the nodes in order to avoid over complication of the work.

### Objectives of the study

At the moment that a message and a node meet, at least two factors can play role in defining the reaction: the characteristics of the node and the characteristics of the message. The current study has only taken the latter into consideration. Can we explain how a message would be treated in the network by studying its characteristics? This forms the questions of the study.

Facebook provides a practically helpful platform to perform such investigation. It’s also one of the top three most visited websites of all (Hennig-Thurau et al. 2013). In this study a Facebook *post* published on a public page is considered as a message. Network behaviors are observed in two forms: (1) the number of times a Facebook post was *liked* (by pressing the like button) or it was *shared* (by pressing the share button) and (2) the type of comments that the Facebook users wrote on the posts.

Number one is about Facebook’s predesigned ways of reacting to a post. Number two is about the way people react to a post by commenting on it. Comments are clearly more difficult to be studied than the number of shares and likes because one would need to go through a complicated process of coding and analysis to define different types of comments.

To be more specific about different types of comments we can categorize them to *positive*, *negative*, and *neutral* comments. Later it will be explained how we systematically classified user comments into more detailed categories. As for the Facebook posts, we would need to classify them into different types. Broadly speaking, this study classifies the posts based on their *form, subject*, *form of expression*, *source*, and the existence of *praise* and *criticism*. Later it will be explained how we categorized the Facebook posts into more detailed types.

**Fig. 1:**
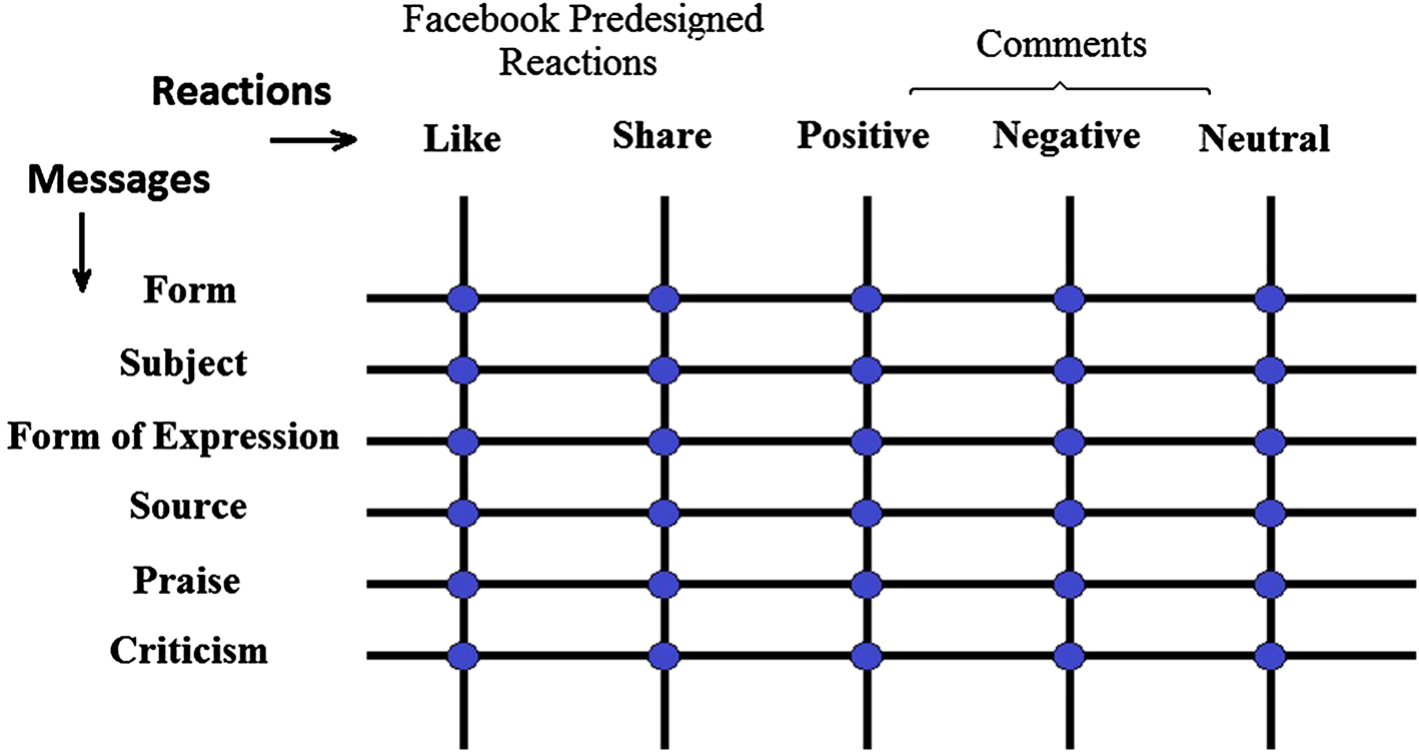
General types of messages and types of reactions toward them

This study will look for significant relations between the type of messages on Facebook and the kind of reactions they would receive based on the general categories below (Fig. 1).

#### Q1: In a given Facebook society what characteristics of the posts in terms of form, subject, form of expression, source, and existence of praise and criticism are in significant relations with the number of times those posts are liked or shared?

The number of *likes* is important in the fate of the messages on Facebook for different reasons. A higher number of likes may be the indication of a higher attention to the post. Also when a post is liked it is automatically shown to other Facebook users, suggesting them to check out the post because their *friend* has liked it. Some studies have shown relations between the number of likes and people’s attention to specific content e.g. loyalty to a brand (De Vries et al. 2012).

Studies in the field of marketing have shown positive relations between liking a brand on Facebook and brand evaluations (Beukeboom et al. 2015). If there are significant relation between some characteristics of Facebook posts and their higher chance of being liked by Facebook users, those characteristics could be very interesting for academicians. They can be also practically useful for marketers and advertisers as characteristics that help the message be seen by a larger number of people in the network.

Also if significant relations are found between the characteristics of the messages (here Facebook posts) and the number of times they are shared, one could try to use them to explain why some materials are shared more often than others. We do not expect that discovering any number of significant relations can enable us to fully explain or predict the number of times people would share a message. However, if we can find some relations, then one can argue that looking for this type of relations is a reasonable method to study the flow of different messages through a social network.

#### Q2: In a given Facebook society what characteristics of the posts in terms of form, subject, form of expression, source and existence of praise and criticism are in significant relations with positive, negative and neutral characteristics of the comments that are written on them?

Other than liking and sharing, people can choose what to write on a post as a comment. Both Facebook posts and the comments written on them by people can be defined by their characteristics. After that the relations between these sets of characteristics can be studied. In a broad sense the comments can be positive, negative or neutral. However, they can be classified based on more detailed characteristics which will be explained.

As an explorative study this research has questions rather than hypotheses. A hypothesis studies predicted relations between two specific variables. Explorative studies may study hundreds of relations between several variables. The current study will test some 1300 relations. Therefore, hypotheses are not appropriate for this study.

## Methods

### Data gathering

Possible audiences of a Facebook public page are the members of those pages. We looked for the most popular Facebook pages in terms of the number of their members and eventually chose 41 Farsi pages. Farsi is the language spoken mostly in Iran, Afghanistan and Tajikistan and the chosen pages were Iranian ones. This benefits the study at least in two ways.

First, it limits the number of popular pages and makes the selection possible. Second, even if there are significant relations between the characteristics of the messages and the reactions toward them, we cannot just assume those relations would be universal.

The probable relations can be specific to a network and its audiences. As an explorative research it would be better to limit the audiences to smaller geographical and cultural areas. Performing several studies on different societies and networks would enable future studies to draw out possible *local* and *universal* relations.

On Facebook pages, the admin or admins of the page post different messages and each message receives a specific number of likes, shares and comments. This study needs (1) the posts (in any format such as text, image or video), (2) the number of its likes and shares, as well as (3) the text of the comments people have written on them. In order to gather this data in a large scale a software application had to be designed.

We explained the idea to a professional computer programmer and asked him to code a software application that could collect Facebook posts and their related information. Once the application was ready we gave the URL of the 41 chosen pages to the application and it copied hundreds of thousands of the messages and comments on those pages. Later 591 posts and 770 comments were randomly chosen. Now it was time for the data to be processed and prepared for data analysis.

### Data processing

The number of likes and shares that a Facebook post receives are countable variables. However, looking for significant relations between the characteristics of the messages and the characteristics of the comments written on them could be done only after we define such characteristics.

1. Characteristics of the messages (Facebook posts)

We tried to define the characteristics of the messages with the help of two colleagues. We started observing many of the gathered messages and looked for any common attributes between the 591 posts in terms of *form*, *subject*, *form of expression*, *source* and the existence of *praise* and *criticism*.

Some characteristics were easy to see and define. For instance, some posts included a picture and some did not. Some included an advertisement while some didn’t. Some characteristics were in common between many posts but they were not easy to define, such as being nostalgic. They needed to be discussed, studied and redefined by the research team. Once a first draft of the common attributes was proposed the list needed to be examined and redefined.

Three researchers cooperated in the long and challenging process of finding and defining the common characteristics of the posts. First by observing many messages a list of 64 characteristics were proposed with their preliminary definitions. Then the researchers coded a number of posts individually and after comparing the results they revised the operational definitions to make them more practical and less ambiguous.

This process was repeated several times until the difference between the results of the work of the researchers was less than 2%. The leading researcher then made the final decision for the 2%. Also 23 of the initially proposed characteristics were omitted from the list, either because their operational definitions could not get clear enough or because there were not enough cases in the chosen Facebook posts to lead to any statistically significant results.

Eventually the 591 Facebook posts were coded for *having* or *not having* any of 46 characteristics. This included making more than 27,000 decisions. Sometimes these decisions had to be made several times by the same or different coders in an attempt to provide reliable results.

Also Cohen’s Kappa was calculated to insure interrater agreement and the reliability of the coding results.

$${\text{k}} = \left( {\Pr \left( {\text{a}} \right) - \Pr \left( {\text{e}} \right)} \right)/\left( {1 - \Pr \left( {\text{e}} \right)} \right)$$

The calculated Kappa was over 0.86, which shows excellent interrater agreement.

The final characteristics are as follows:

#### *Form*

links, texts, photos of famous people, pictures (excluding the photos of famous people), video clips, music.

#### *Subject*

psychological issues, political issues, economic issues, religious (or anti-religious) issues, social and cultural issues, scientific issues, relations with the opposite sex, family relationships, environmental issues, literary materials, news, request for like or share, questions or request for comments.

#### *Form of expression*

disappointing materials, warning, expression of sorrow or regret, emotional materials, spiritual emotions, advice, nostalgic materials, jokes, satire, poems or literature, insult, hopeful materials, reasoning and analysis, artistic materials, aphorism, advertisement.

#### *Source*

quotes from famous people, quotes from religious texts, quotes from sources, personal opinions.

#### *Praise*

admiration of the current politicians or political system of Iran, admiration of individuals.

#### *Criticism*

criticism of individuals, criticism of a social group, criticism of the current politicians or political system of Iran, criticism of the politicians or political system of other countries, criticism of Iran or Iranians.

During the coding process, each characteristic was treated as an individual variable to be checked. For instance, coders did not ask themselves a question such as the following: Is this post political or economic? They would check the post to see if it contained economic issues regardless of their decision if it included political contents.

2. Network Behavior of the Nodes (User comments on Facebook posts)

The 770 comments were coded in a similar way to the process used for the posts. Similar to what was done before, we made sure coding process and its results were reliable. Cohen’s Kappa being over 0.84, interrater agreement was proved to be reliably high.

The final list of characteristics of the network behaviors includes 24 items. Here we did not consider the types of issues (say economic, political, etc.) in defining the characteristics of the comments. Network behaviors are about the type of *reactions*. Reactions could be broadly categorized and positive, negative or neutral.

The final list of characteristics for the network behavior of the nodes is presented below.

#### *Positive*

expressing agreement, defending the post against another commenter, expressing gratitude, expressing positive emotions.

#### *Negative*

questioning the post, insulting the admin, blaming the admin for uploading such a post, insulting or making pungent remarks, showing partial disagreement.

#### *Neutral*

adding to what was presented in the post, making recommendations, correcting the post’s mistake, joking with the admin of the page, answering questions, raising a question or request, trying a game or enigma, answering a multiple choice question, inviting others to see the post, reacting to another commenter, reasoning and analysis, joking and making sarcastic comments, laughter, expressing emotions, advertising or irrelevant.

This part of the coding process also included finding, defining and redefining the attributes and more than 20,000 instances of decision-making and they had to be done more than once.^1^

## Results

In this section the results of the statistical analysis of the relations between the proposed sets of data will be reported.

### Results regarding the first question

The first question concerns the relations between the characteristics of the Facebook posts and the number of *likes* and *shares* they received. To make the statistical analysis possible the posts had to be divided to at least two groups based on the number of likes and shares they received. If a post was *liked* by 1800 people or less it was coded as *Low* and if more than 1800 people liked a post it was coded as *high*. For this section of the study 491 posts were taken into consideration, among which 245 posts fell under the group with *low* number of likes, and 246 posts received *high* number of likes.

On Facebook the number of people who share a post is always less than those who *like* it. If a post was *shared* by 200 people or less, it was coded as *Low*. If more than 200 people shared a post it was coded as *high*. For this section of the study 470 posts were taken into consideration,^2^ among which 240 posts fell under the group with *low* number of times being shared and 230 posts fell under the group with *high* number of times being shared.

Statistical analysis of the relations between the number of people who liked and shared a Facebook post and the existence of any of the 46 characteristics resulted in 30 significant relations. Pearson’s Chi-square test was used to see how significant the results were, and only the ones with a level of significance equal to or higher than 95% were reported.^3^ Many of the reported relations were much more than 95% reliable. These relations have been summarized in Table 1.

**Table 1:** Significant relations between the characteristics of the posts and the number of *likes* and *shares*

Characteristics of the posts	Facebook predesigned reactions			
	Like		Share	
Form			Pictures (excluding the photos of famous people)	40%
Subject	Questions or request for comments	−28%	Questions or request for comments	−61%
	Psychological issues	−41%	Religious (or anti-religious) issues	35%
	Family relationships	39%	Request for like or share	−42%
	Relations with the opposite sex	50%	Social and cultural issues	22%
	Request for like or share	−35%	Literary materials	52%
Form of expression	Satire	55%	Jokes	−50%
	Advertisement	−53%	Satire	43%
	Artistic materials	44%	Advertisement	−41%
	Emotional materials	42%	Nostalgic materials	−44%
	Reasoning and analysis	−26%	Emotional materials	23%
			Advice	30%
			Reasoning and analysis	30%
			Disappointing materials	31%
Source			Personal opinions	60%
			Quotes from famous people	41%
			Quotes from sources	30%
Praise	Admiration of individuals	32%	Admiration of the current politicians or political system of Iran	−66%
Criticism			Criticism of Iran or Iranians	79%

The table shows how existence of specific characteristics in the posts has increased or decreased the probability of receiving a high number of *likes* and *shares*. For instance, there was a significant relation between the existence of satire in the posts and the number of people who *liked* it. In 70.9% of the cases where satire existed in the post, a high number of people pressed the Like button, while when it didn’t exist, only in 45.7% of the cases high number of people did so.

This indicates that there was a direct relation between the existence of this characteristic in the post and its likelihood to receive *likes* from the people. The level of significance for Pearson’s Chi-square test was more than 99.9%. Table 1 presents the results in a different way. The existence of satire in the posts increased the probability of being *liked* by 55%.

The existence of some characteristics had an invert effect on the probability of a post being *liked*. For instance, if analytical and reasoning-based materials existed in a post it had 26% less chance that its audiences pressed the *like* button. The rest of the numbers can be seen in the table.

The analyses aimed at answering the first question of the study show that characteristics related to the materials’ *subject*, *form of expression* and existence of *praise* were in significant relations with the likelihood of the posts being liked. They also showed that the materials’ *form*, *subject*, *form of expression*, *source*, and existence of *praise* and *criticism* were in significant relations with the likelihood of the posts being shared (Table 2).

**Table 2:** Relations found between general types of messages and number of *likes* and *shares*

Facebook post	Facebook predesigned reactions	
	Like	Share
Form		✓
Subject	✓	✓
Form of expression	✓	✓
Source		✓
Praise	✓	✓
Criticism		✓

### Results regarding the second question

The second question concerns the relations between the characteristics of the Facebook posts and the characteristics of the comments written on them. The statistical analysis of the relations between these two sets of characteristics showed 105 significant relations, presented in Table 3.

**Table 3:** Significant relations between the characteristics of the posts and the characteristics of the comments

Facebook post	Comments													
	Positive			Negative				Neutral						
	Showing agreement	Expressing gratitude	Expressing positive emotions	Adding to what was presented in the post	Insulting or making pungent remarks	Showing partial disagreement	Questioning the post	Blaming the admin for posting such a post	Answering	Answering a multiple choice question	Reasoning and analysis	Joking and making sarcastic Comments	Laughing	Expressing emotions
Form														
Photos of famous people	–	–	–	32%	103%	–	–	–	-48%	–	–	–	–	511%
Subject														
Questioning or requesting comments	−80%	−89%	–	−56%	−69%	–	–	–	2406%	6040%	–	–	–	–
News	−66%	−75%	–	78%	199%	–	–	–	−77%	−100%	–	–	–	–
Religious (or anti-religious) issues	128%	–	–	–	–	–	–	–	–	–	–	–	–	–
Social and cultural Issues	118%	65%	−91%	34%	–	–	68%	–	–	−66%	167%	–	66%	−57%
Request for like and share	−100%	–	–	–	–	–	–	–	–	–	–	–	–	–
Political issues	–	–	−70%	79%	113%	–	–	–	−51%	−94%	122%	–	–	−54%
Psychological issues	–	–	–	–	–	–	–	–	125%	–	–	–	–	–
Form of Expression														
Disappointing materials	144%	–	–	83%	–	–	–	–	−61%	−100%	–	–	–	−72%
Warning	110%	–	–	58%	–	–	–	–	–	–	–	–	–	−100%
Expression of sorrow or Regret	224%	–	–	42%	–	–	–	–	−100%	–	–	–	–	–
Emotional materials	–	–	337%	–	–	–	109%	–	–	–	–	−49%	–	226%
Advice	59%	310%	–	44%	–	–	–	–	−90%	−100%	–	−55%	–	–
Jokes	–	–	–	−100%	–	–	–	–	–	–	–	–	–	–
Satire	–	–	–	−38%	–	–	–	–	−63%	–	−80%	98%	163%	–
Hopeful materials	–	–	–	50%	–	–	–	–	–	–	–	–	–	–
Reasoning and analysis	126%	268%	−100%	68%	75%	–	–	–	−55%	−100%	238%	–	–	−79%
Artistic materials	–	–	–	–	–	–	–	–	–	416%	–	–	–	105%
Source														
Quotes from famous people	51%	−57%	–	98%	–	–	–	–	−66%	−100%	–	–	–	–
Quotes from sources	64%	221%	−83%	96%	146%	–	–	–	−75%	−100%	146%	–	–	−73%
Personal opinions	427%	252%	−65%	34%	–	357%	80%	336%	−67%	−93%	–	–	–	−40%
Praise														
Admiration of individuals	–	–	–	–	–	–	–	–	–	–	–	–	–	99%
Criticism														
Criticism of social group	143%	–	–	44%	–	–	–	–	–	–	–	–	–	–
Criticism of individuals	84%	–	–	61%	267%	–	–	–	–	–	–	–	–	−72%

Data presented in Table 3 show, in a similar way, how the probability of specific network behaviors can change with the existence of specific characteristics in the post.

For example as the table shows, there are at least 12 characteristics that their existence in a post increased the probability of *expressing agreement* by the commenter. And there are at least 3 characteristics that their existence in the post decreased such probability.

The results show that if there are quotes from famous people in the post the chance that people show agreement to the post in their comments increases by 50%. For example if in the absence of quotes from famous people, 10% of the commenters expressed agreement, where quotes from famous people existed, 15% of the commenters expressed agreement.

Some of the numbers in the tables are more than 100 which make the results more interesting. For instance the existence of an advice in the post increased the chance that the commenters expressed gratitude by 310%.

The existence of one characteristic in the post could have a relation with several behaviors. The table could be rearranged so that this is seen easier. Table 4 that was extracted from Table 3 shows all the behaviors that changed with the existence of a personal opinion .

**Table 4:** Behaviors in significant relation with the existence of personal opinions in the post

Personal opinions	%
Expressing agreement	427
Expressing gratitude	252
Adding to what was presented in the post	34
Answering	−67
Expressing positive emotions	−65
Expressing emotions	−40
Showing partial disagreement	357
Questioning the post	80
Blaming the admin	336

When a personal opinion existed in the post, the probability that people showed agreement increased by 427% but at the same time the chance that the admin was blamed increased by 336% and the probability of partial disagreement increased by 359%. Understandably personal opinions provoke controversy especially in polarized societies.

The 105 significant relations link *form*, *subject*, *form of expression*, *source* and the existence of *praise* and *criticism* in the Facebook posts with *positive*, *negative* and *neutral* comments written by the users. Table 5 shows what relations were observed between the abovementioned variables.

**Table 5:** Relations found between general types of messages and types of comments

	Comments		
	Positive	Negative	Neutral
Form		✓	✓
Subject	✓	✓	✓
Form of expression	✓	✓	✓
Source	✓	✓	✓
Praise			
Criticism	✓	✓	✓

## Discussion

### The issue of hidden variables

Reviewing the results, we might find some relations hard to explain. For instance, one might ask: why should criticism of social groups increase the probability of expressing agreement from the commenter by almost 150%. Is this result reliable? Two points should be noted in this regard.

1. The levels of significance are high but not a hundred.
  In any research project in which hundreds of relations are statistically studied, no matter how high the level of significance is, a concern would be having few relations occurring by accident. For instance, if the average level of significance in this project is 98%, out of 130 reported results 2 or 3 might be a result of chance rather than robust existing patterns.
  The only way to find out whether or not there were such cases among the results, and if yes, which of the results were due to chance, is waiting for additional studies to provide us with a better understanding of the issue. However, it is unlikely that many false relations exist in the results.
2. Sometimes *what* is more important than *why*
  The results indicate existing relations between two sets of variables and not causal relations between them. We cannot expect all the relations to seem predictable and understandable. Some of the significant results may be in consistence with what we already expect and for some of the results we may not have any explanations. This does not change the fact that there is a very high chance that those patterns are there and the first step to better understand them is to know they exist.
  Even where we don’t seem to understand a relation there may be very good explanations; however sometimes even knowing why a relation exists is not worthy of the effort needed to find it out. The reasons behind a relation between two variables can be very complicated and many factors may be engaged. Sometimes based on the projects’ needs, rather than explaining why specific groups of people act in a specific way, we just need to know how they behave.

### The black box of human brain

The behaviors studied here are the results of human reactions. When a person responses to the stimulus of a message, deep “underlying impulses” (Bandura 1971) can be involved in their reaction. To understand those underlying impulses studying human behavior, as well as, people’s static attributes (say gender, age class, etc.) and dynamic attributes—say opinion, interest, etc.—(Bouanan et al. 2016) can be helpful.

Since the acquired information “serves as a guide for action” (Bandura 1974) deeper understanding of the interactions between people and information can be acquired through understanding people’s psychology and behavior. Moreover, to better understand the reactions of the nodes in a network we need to understand their individual and social experiences; as people are “agents of experience” (Bandura 2001).

**Fig. 2:**
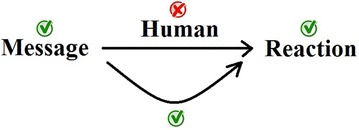
Disregarding human factor in studying the relations between messages and reactions

However, as mentioned before this study intentionally disregarded the characteristics of the human factor and instead looked at their reactions (Fig. 2).

**Fig. 3:**
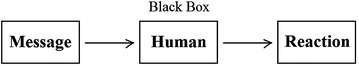
Disregarding human factor in studying the relations between messages and reactions

In behavioral studies sometimes human brain is considered as a black box that we might not be aware of the processes within it. However, interaction between communication and behavioral studies can help us develop more comprehensive methods to investigate, understand and even predict human reactions in confrontation with different types of messages (Fig. 3).

## Conclusions

Since this is an explorative work, trying to pave the way for future studies, at least parts of its results are general findings that can inspire future studies. However, it has very detailed and specific outcomes as well.

The main outcomes of the study can be introduced as follows:

1. By ignoring other factors and only studying the characteristics of the messages running within given communities on Facebook, researchers can find factors in significant relation with the number of times the messages are liked.
  This might be true in other networks too. The results of the study indicate that it is worth the expenses and the effort to conduct large scale studies to see what characteristics in a message can attract more *likes* on Facebook or more attention on other networks.
2. It may be hard to generalize this to all communities within Facebook, but the results of the study among Iranian pages show that the following features, if existing in a Facebook message, had negative impact on its chance of receiving likes from the users:
  asking questions or requesting comments
  advertisements
  psychological issues
  request for like or share
  reasoning and analysis
  On the other hand, the existence of the following features increased the chances of receiving likes by a Facebook post.
  satire
  artistic materials
  family relationships
  relationship with the opposite sex
  emotional materials
  admiration of individuals
  We have also acquired numbers telling us how much the chances of receiving likes would increase or decrease. This information can have practical values for campaigners and companies. Also the results can be of value for those interested in conducting similar but more comprehensive/focused studies or studies among different communities.
3. Similar to what was mentioned in the outcome number 1, we have shown that by exploring the characteristics of the messages we can find features increasing or decreasing their chances of being shared with others. In fact, the results of this study shows that the idea of calculating the probability of a message being distributed between people can have practical values.
4. Similar to what mentioned in the outcome number 2; we have a list of features that their existence in a message can increase or decrease its chances of being shared with others. We also have numbers showing to what extent those features can change the probabilities. Since the list has been already provided in tables, they will not be repeated here. This list can be used by campaigners, advertisers or researchers.
5. This study put forward the concept of network behaviors and argued that the characteristics of messages can define the way they are treated on a network and proposed that these relations could be studied in a detailed level. The fact that several significant relations were found between the types of Facebook materials and the way people reacted to them proves this approach practical.
  Other studies can target specific networks, specific communities and specific types of materials (say brands, political issues, etc.) and use the method proposed in this research to study what characteristics can increase the probability of people reacting in desired ways.
6. We have found a long list of significant relations between the characteristics of the messages and the way people react to them. We also have numbers indicating how much a specific characteristic have increased or decreased the likelihood of a reaction. Campaigners, advertisers and researchers may pick specific characteristics and use them in their projects or studies.

As a preliminary work we tried to introduce a way to approach the study of flow of information in a digital social network and examine the practicality of that approach. Since several significant relations were drawn, it seems to be clear that this approach is practical and with several future studies, there would be hope to reach a point where we have a list of practical relations that tell us why different messages travel differently in the network.

To be ambitious maybe future works give us the power to predict the fate of a message if released in the network. In such an ideal situation we may be capable of predicting how long a message would keep moving before it stops reaching new people; how many people it would reach; what types of people it would reach, etc.

As mentioned before, neither network behaviors nor message characteristics had to be defined in the way we did in this study. Longer, more practical, socially more sensitive lists of characteristics and behaviors, proposed by future studies may result in much more practical guidelines for us to understand why specific messages travel in the network the way they do.

### Other applications

Other than understanding how network flow of information works, information can be drawn from the results to better understand the society of Iranian Facebook users—if not generalizable, at least as clues for future studies. For instance the results show that *disappointing materials* have increased the probability of expressing agreement by 150%. Also *expression of sorrow or regret* has increased the probability of showing agreement by 224%. For a study focused on that society, this information may be meaningful.

Also other studies—not concerned with flow of information—may use the method used in this study to understand people. Other scholars may develop ways of exploring people by analyzing their reactions to different types of content.

## Footnotes

1 In the recent years automated methods of text analysis including Linguistic Inquiry and Word Count (LIWC) have improved. Since the process of manually coding posts is extremely time-consuming, the current study was able to analyze a limited number of posts and comments which can be considered a limitation of the study. Automated methods have allowed research projects to consider large amounts of text and as a result have significantly improved generalizability of the studies. The current research did not employ such automated systems for a number of reasons, as follows:

- While Farsi dictionaries have improved, the structure of the language makes it difficult to perform highly reliable automated works, especially on informal texts. In informal environments a significant number of words are written differently from their dictionary form.
- In informal English, *he is going to* becomes *he’s gonna.* Such changes happen to almost every Farsi verb and the changes are major: *bekhaaheem* becomes *bekhaaim* and *bekhaahand* changes to *bekhaan*. Considering the intentional misspelling of words by very young generations (who tend to write only the one or two initial letters of the words) the problem is even more serious. This is why, although Farsi dictionaries have significantly improved and they are employed in many applications, when it comes to informal texting (such as in cell-phone conversations) they are next to useless.
- Farsi uses Perso-Arabic alphabet which is based on Arabic script. However, many Facebook users use English alphabet to type Farsi; similar to the sample Farsi words presented above to show how informal Farsi is written differently from formal Farsi. Farsi dictionaries do not work with the texts typed in this way, which is called Finglish (Farsi + English).
- In our analysis we also considered non-written characteristics of the posts such as photos.
- We tried to detect a long list of characteristics/types in the posts and comments such as aphorism, opinions, reasoning, spiritual emotions, etc. The existence of such features is very hard for a robot to detect.

2 The analyzed data was gathered using a software application. The application was not able to retrieve data regarding the number of likes and shares for over 100 of the selected posts.

3 To show the status of correlations between the variables in this study, we used Chi-square test. Here, Chi-square as a nonparametric test was used instead of parametric tests such as independent *t* test. One reason for this is the fact that the variances between the two comparison groups were different. Also in this study we were dealing with non-normal distribution of data, and more importantly the variables were all nominal.
